# A Sargassum-associated phage slows Vibrio alginolyticus persistence on macroalgal surfaces

**DOI:** 10.1099/mgen.0.001873

**Published:** 2026-11-10

**Authors:** Zhiheng Zuo, Huaijin Jiang, Peng Zhu, Qian Zhang, Mingrui Li, Ligong Zou, Rui Zhang, Yunlan Yang, Yufeng Yang

**Affiliations:** 1 Department of Ecology, Jinan University/Southern Marine Science and Engineering Guangdong Laboratory (Zhuhai), Guangzhou, 510632, PR China; 2 Archaeal Biology Center, Synthetic Biology Research Center, Shenzhen Key Laboratory of Marine Microbiome Engineering, Key Laboratory of Marine Microbiome Engineering of Guangdong Higher Education Institutes, Institute for Advanced Study, Shenzhen University, Shenzhen, 518055, PR China

**Keywords:** biofilm inhibition, macroalgal holobiont, phage, *Vibrio alginolyticus*

## Abstract

Macroalgal surfaces harbour dense and metabolically active microbial consortia in which *Vibrio alginolyticus* exhibits marked ecological plasticity, spanning from mutualistic to opportunistic lifestyles. In these structured biofilm habitats, phages play a central role in shaping bacterial population structure and metabolic interactions. Here, a lytic phage vB_ValP-R39Z (R39Z) and its *V. alginolyticus* host were isolated from the surface of *Sargassum hemiphyllum*. Phage R39Z displays a podovirus morphotype, with a latent period of ~50 min and a burst size of 109 p.f.u. per cell, a narrow host range restricted to its original host, and stability across environmentally relevant conditions (pH 7–9 and 4–35 °C). R39Z harbours a 71.8 kb dsDNA genome (37.7 mol% G+C), encoding 83 ORFs, and is distinct from established genera, representing a previously unclassified genus. Functionally, R39Z significantly reduced biofilm biomass *in vitro*, and in an ex vivo assay using *Sargassum* blades, it reduced the viable cell abundance of *V. alginolyticus* ZS10 on blade surfaces by ~99%, as determined by c.f.u. enumeration. These findings demonstrate that R39Z efficiently disrupts biofilm formation and suppresses *Vibrio* colonization on macroalgal surfaces, underscoring its ecological relevance and potential for targeted biocontrol in algal-associated microbiomes.

**Impact Statement**

Macroalgal surfaces represent densely colonized microbial habitats, where *Vibrio alginolyticus* can shift between beneficial association and opportunistic overgrowth, with consequences for algal health and aquaculture. However, most evidence for phage activity in macroalgal microbiomes remains sequence-based, and experimentally characterized phage–host systems from macroalgal surfaces are still scarce. In this study, a lytic phage isolated from *Sargassum* specifically suppresses *V. alginolyticus* both in biofilms and on fresh macroalgal blades. These findings provide direct experimental evidence that naturally occurring phages can regulate bacteria on macroalgal surfaces, highlighting their potential as targeted biocontrol agents in macroalgal-associated microbiomes.

## Data Summary

1. The complete genome sequence (GenBank: PX585279) and raw sequencing reads (SRA: SRR38202926; BioSample: SAMN57386513) of phage vB_ValP-R39Z have been deposited in National Center for Biotechnology Information (NCBI).
2. The 16S rRNA gene sequences of all 20 bacterial strains used in this study, including the host strain *Vibrio alginolyticus* ZS10 and all strains tested in host range assays, have been deposited in NCBI GenBank under accession numbers PX963265 (ZS10) and PZ306299–PZ306317 (the remaining 19 strains). A complete strain-to-accession mapping is provided in Table S1, available in the online Supplementary Material.
3. Whole-genome sequencing reads and draft genome assemblies for five *V. alginolyticus* strains (SS6, ZS7, ZS8, ZS9 and ZS10) used in comparative genomic analyses have been deposited in NCBI under BioProject PRJNA1456041. The raw reads are available under SRA accession numbers SRR38204978–SRR38204982, and the draft genome assemblies are available under GenBank WGS accession numbers JBXPSQ000000000, JBXPSP000000000, JBXPSO000000000, JBXPSN000000000 and JBXPSM000000000.

## Introduction

Macroalgal surfaces provide nutrient-rich microhabitats that promote epiphytic biofilm formation and support surface-associated bacterial communities [1]. Biofilm formation, mediated by extracellular polymeric substances (EPSs), enhances bacterial retention and modulates nutrient exchange at the macroalgal surface, influencing macroalgal growth, stress tolerance and disease susceptibility [2]. Within these epiphytic biofilms, *Vibrio* spp. are recurrent and often abundant components of the surface microbiome, thriving in the nutrient-rich and spatially structured microhabitat of the macroalgal phycosphere. In some cases, particle-attached *Vibrio* cells may constitute up to ~70% of the surface-associated bacterial community [3].

*Vibrio alginolyticus* demonstrates remarkable ecological versatility. It can act as a symbiotic or mutualistic associate or turn to an opportunistic pathogenic lifestyle, depending on the host and surrounding environmental conditions [4]. A recent study has indicated that *V. alginolyticus* strain X-2 could mitigate bleaching disease in the brown alga *Saccharina japonica*, exerting probiotic-like effects that support macroalgal health and stress tolerance [5]. However, during the outbreaks of ‘Baotou disease’ in the red alga *Gracilariopsis lemaneiformis*, *V. alginolyticus* has been identified as the dominant pathogenic bacterium, indicating its conditional virulence and environmental sensitivity [6]. This ecological persistence suggests that *Vibrio* populations on macroalgal surfaces are likely shaped by multiple regulatory factors that determine whether they persist as beneficial symbionts or shift towards pathogenic overgrowth.

Among the potential regulators of surface-associated *Vibrio*, phages are increasingly recognized as important modulators of bacterial mortality, community structure and microbial turnover [7]. In marine systems, lytic phages contribute to the viral shunt by lysing bacterial cells and releasing organic matter and nutrients into dissolved pools, thereby supporting microbial recycling [8]. Phages can also influence host physiology more directly through metabolic reprogramming during infection, including through auxiliary metabolic genes (AMGs). Recent viromic studies have revealed a large number of novel phages in macroalgal-associated microbial consortia, indicating that phages are likely integral yet still underappreciated components of the macroalgal holobiont [9]. However, these insights remain largely sequence-based, and experimentally tractable phage-host systems isolated from macroalgal surfaces remain scarce. As a result, key aspects of macroalgal-associated phage biology, including host specificity, infection dynamics and the ecological significance of phage-mediated suppression of surface-associated bacteria, remain poorly understood.

In this study, a lytic phage infecting *V. alginolyticus*, derived from the surface of *Sargassum hemiphyllum*, was isolated and characterized. Plaque morphology, electron microscopic morphology and infection dynamics were investigated to examine the phage’s biological traits. The genome was sequenced and analysed to characterize its coding potential, taxonomic placement and metabolic features. Potential bacterial determinants of the narrow host range were examined by comparing surface polysaccharide loci and anti-phage defence systems among five closely related *V. alginolyticus* isolates. In addition to evaluating the effects of the phage on biofilm formation and disruption *in vitro*, an ex vivo colonization assay using fresh *Sargassum* blades was performed to evaluate its ability to inhibit surface-associated *Vibrio* populations in a native-like phycosphere. By integrating biological, genomic and ecological evidence, this study aimed to explore how a macroalgal-associated phage may contribute to microbial regulation within the macroalgal holobiont and inform future phage-based biocontrol strategies in coastal aquaculture.

## Methods

### Isolation and purification of phage

The host strain *V. alginolyticus* ZS10 and phage were isolated from the blade surface of *S. hemiphyllum* collected in a *Sargassum* aquaculture area near Guishan Island, China (22.12° N 113.89° E) (Fig. 1). To collect *Sargassum*-associated micro-organisms and phage, fresh blades were gently rinsed with sterile artificial seawater to remove loosely attached particles, and the blade surfaces were swabbed using sterile cotton swabs. The cotton swabs were transferred into 20 ml of sterile artificial seawater and stored at 4 °C until further analysis.

**Fig. 1.**
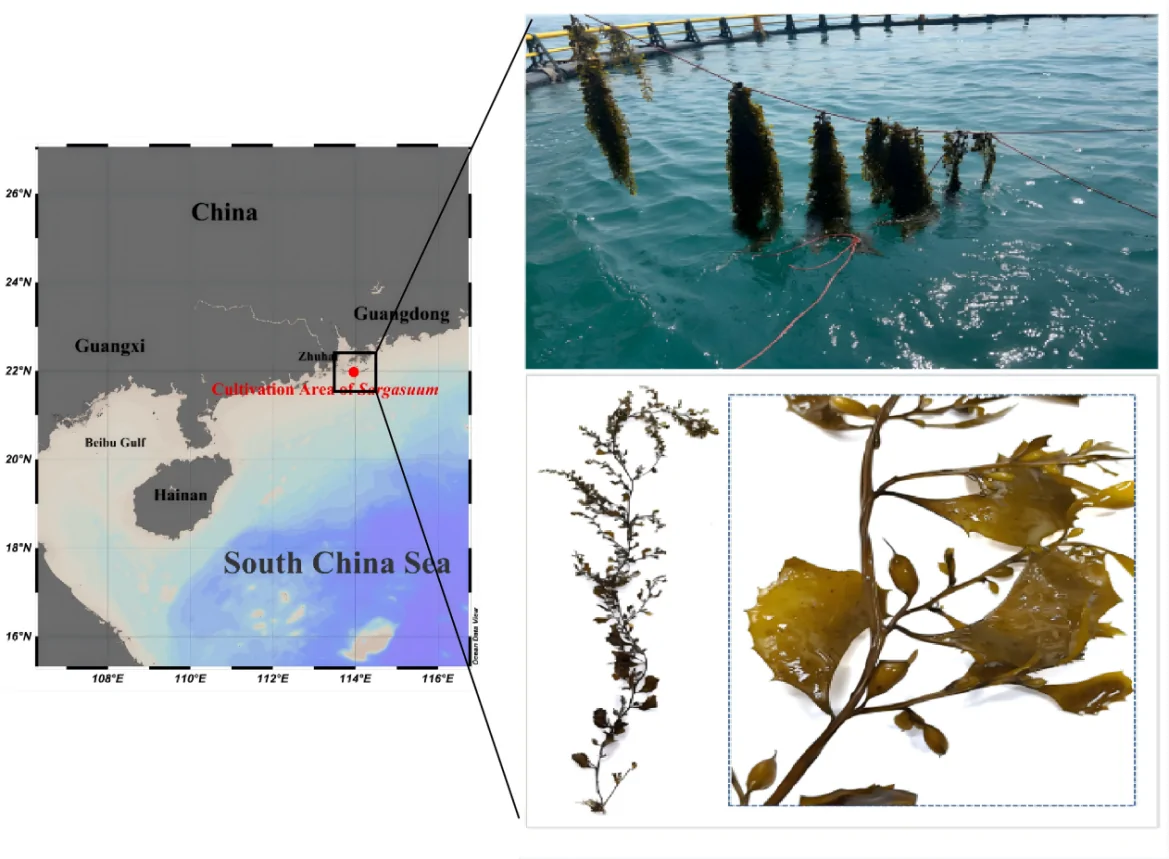
Isolation environment of *S. hemiphyllum* used for the isolation of *Vibrio* and its phages. Samples were collected from a cultivation site near Guishan Island, Zhuhai, China (red dot on the regional map). The photograph shows the cultivation system and a close-up view of representative algal blades used for phage and bacterial isolation.

To isolate the host strain, cotton swabs were shaken in sterile seawater at 28 °C and 170 r.p.m. for 30 min. The blade surface suspension was serially diluted, spread onto marine agar 2216E and incubated at 28 °C for 24 h, and single colonies were restreaked three times to obtain a pure culture. The strain was identified by blastn analysis of its 1,436 bp partial 16S rRNA gene sequence (accession number PX963265), which shared 99.51% identity with the type strain *V. alginolyticus* ATCC 17749^T^ over 1,422 bp (99% query coverage). Species-level assignment was further supported by FastANI v1.34 comparison of the ZS10 draft genome (accession number JBXPSM000000000) against the *V. alginolyticus* ATCC 17749^T^ reference genome (GCF_000354175.2), yielding an average nucleotide identity of 98.40% and an alignment fraction of 93.64%. Phage isolation was performed by centrifuging the blade surface suspension at 4 °C and 8,000 ***g*** for 10 min, and the supernatant was filtered through a 0.22 µm sterile membrane. Subsequently, 10 ml of the filtrate was mixed with 10 ml of exponentially growing *V. alginolyticus* ZS10 in marine broth 2216E and incubated at 28 °C and 170 r.p.m. for 24 h. Phage was isolated and purified by the double agar overlay plaque assay [10]. Briefly, the resulting enrichment culture was then filtered (0.22 µm), serially diluted in autoclaved SM buffer (50 mM Tris-HCl, 0.1 M NaCl, 8 mM MgSO_4_; pH=7.5) and mixed with exponential-phase host cells to prepare double agar overlays. Plates were incubated at 28 °C and monitored for plaque formation. Clear, discrete plaques were picked into SM buffer and stored at 4 °C. At least five rounds of plaque purification were performed to obtain clonal phage populations. Plaque size and morphology were recorded with a plaque-imaging camera (SONY ILCE-7RM3A).

### Preparation of high-titre phage suspensions

Purified phage suspensions were filtered through 0.22 µm membranes to remove residual cells and debris, inoculated into 20 ml exponential phase *V. alginolyticus* ZS10 and scaled up to a 1 l culture. To facilitate phage detachment from cell debris, NaCl was added to the mixture to a final concentration of 100 g l^−1^ and incubated at 4 °C for 1 h. After removing host debris by centrifugation (8,000 ***g***, 30 min), the lysate was treated with DNase I and RNase A (1 µg ml^−1^) for 30 min at room temperature to remove extracellular genetic material. The suspension was filtered through a 0.22 µm membrane and then concentrated via PEG 8000 (100 g l^−1^) with overnight incubation at 4 °C. The precipitate was collected by centrifugation (10,000 ***g***, 4 °C, 60 min), resuspended in 5 ml of SM buffer and treated with chloroform (1 : 1 v/v) to remove residual impurities. The resuspended phage was then purified by CsCl equilibrium gradient centrifugation with layers of 1.3, 1.5 and 1.7 g ml^−1^ at 200,000 ***g***, 4 °C for 6 h with an Optima L-100 XP ultracentrifuge (Beckman Coulter, Brea, CA, USA). Phage particles were recovered from the 1.5 g ml^−1^ band and desalted by dialysis using 30 kDa ultrafiltration tubes (Millipore, MA, USA) for subsequent experiments, such as phage morphology analysis and nucleic acid extraction.

### Transmission electron microscopy

Phage morphology was examined by transmission electron microscopy (TEM) using negative staining [11]. A 3 µl aliquot of the dialyzed phage suspension was placed onto a gold-sputtered copper grid and allowed to adsorb for 3 min at room temperature. Excess liquid was removed with filter paper, and the grid was sequentially stained with 2% (w/v) phosphotungstic acid, with each staining step followed by air-drying for 3 min. Images were acquired with a transmission electron microscope (JEOL, Japan).

### Chloroform sensitivity assay

Chloroform sensitivity was determined by statistically comparing p.f.u. titres after exposure to different chloroform concentrations. Phage suspensions (1 ml, 2×10^9^ p.f.u. ml^−1^) were divided into four groups and supplemented with 0, 20, 200 or 400 µl ml^−1^ chloroform, gently mixed and incubated protected from light for 30 min. After centrifugation (5,000 ***g***, 4 °C, 5 min), the supernatants were collected, and phage titres were determined using the double agar overlay plaque assay. Comparative p.f.u. values were used to evaluate the impact of chloroform on phage infectivity and to infer the presence or absence of lipids associated with the virion.

### Host range determination

Host range was assessed by the spot assay [12]. Host range analysis was tested against 35 bacterial strains, including 21 *Vibrio* isolates and additional non-*Vibrio* strains derived from aquaculture, marine and public-health-related environments. Briefly, exponential-phase bacterial cultures (1 ml) were mixed with 5 ml marine agar 2216E (0.5% agar) and overlaid onto marine agar 2216E plates. After the overlays had solidified at room temperature, 5 µl of phage suspension (10^9^ p.f.u. ml^−1^) was spotted, and the plates were incubated at 28 °C for 12 h. Additionally, to assess the lytic sensitivity, tenfold serial dilutions of the phage suspension (10^2^–10^8^ p.f.u. ml^−1^) were spotted onto lawns of each susceptible strain identified in the initial spot assay.

### One-step growth curve

A one-step growth experiment was conducted to characterize the lytic cycle, focusing on latent period and burst size as primary metrics [13]. Various multiplicities of infection (MOIs) were tested to identify the optimal condition for phage propagation (Table S2), and the details of the MOI analysis are provided in Text S1. For the assay, the phage and *V. alginolyticus* ZS10 were mixed at the optimal MOI (0.01), allowed to adsorb at room temperature for 5 min and centrifuged (8,000 ***g***, 4 °C, 5 min) to remove un-adsorbed phages. The pellet was resuspended in 1 ml of marine broth 2216E and transferred into 100 ml of marine broth 2216E (T_0_=0), followed by incubation at 28 °C. Aliquots (100 µl) were sampled every 10 min and titrated by the double agar overlay plaque assay. Three biological replicates were performed, and the mean was reported.

### Thermal and pH stability

Thermal and pH stability of the phage were evaluated by monitoring changes in p.f.u. under different temperature and pH conditions [14]. Phage suspensions (10^8^ p.f.u. ml^−1^) were incubated at 4 °C, 25 °C, 35 °C, 45 °C, 55 °C and 65 °C, with samples taken at 0, 3, 24 and 48 h. For pH stability, SM buffer was adjusted to pH 2–12 using HCl or NaOH, and diluted phage suspensions (10^8^ p.f.u. ml^−1^) were added, and samples were collected at 0, 3, 24 and 48 h. Three biological replicates were prepared per temperature and pH, and phage titres were determined as described above.

### DNA extraction and sequencing

Phage DNA was extracted using the phenol–chloroform–isoamyl alcohol method [15]. Briefly, purified phage solution was treated with proteinase K (100 mg ml^−1^), 100 µl of SDS solution (10% w/v) and 10 µl of EDTA (0.5 M, pH 8.0) at 55 °C for 3 h, followed by three rounds of phenol–chloroform–isoamyl alcohol (25 : 24 : 1) extraction and one chloroform–isoamyl alcohol (24 : 1) wash. DNA was precipitated with isopropanol at −20 °C overnight, washed twice with 70% ethanol, air-dried and resuspended in Tris–EDTA buffer. DNA quantity and purity were assessed by gel electrophoresis and NanoDrop (A260/A280=1.8–2.0). Library construction and whole-genome sequencing were performed by Shanghai Hanyu Biotechnology Co., Ltd. (China). The sequencing library was prepared using the NEBNext^®^ Ultra^™^ DNA Library Prep Kit (NEB, USA) and sequenced on the Illumina HiSeq 4000 platform.

### Phage genomic characterization

Raw paired-end Illumina reads (150 bp) were quality-filtered and adapter-trimmed using fastp v1.1.0. Host-derived reads were removed by mapping against the host genome using BWA v0.7.17, and unmapped read pairs were retained using SAMtools v1.17. The remaining reads were *de novo* assembled using MEGAHIT v1.2.9 [16]. After assembly, genome termini and packaging mechanisms were inferred with PhageTerm v1.0.2 [17]. ORFs were predicted with GeneMarkS [18]. Predicted ORFs were annotated against the PHROGs database using MMseqs2 (e-value ≤1×10⁻⁵; ≥50% query coverage) and assigned to PHROG functional categories [19]. The potential functions were further supplemented by VirSorter2-DRAM-based identification of AMGs and associated pathways, together with InterPro analysis of conserved domains and functional sites [20, 21]. tRNA genes were detected with tRNAscan-SE v2.0 (http://lowelab.ucsc.edu/tRNAscan-SE/) [22]. Key gene products were modelled with AlphaFold (https://alphafold.com/), model confidence was assessed by pLDDT, and structures were visualized in PyMOL v2.3.0 to examine conserved regions and potential functions [23, 24]. Potential virulence and antibiotic resistance genes were screened with the Virulence Factors Database (VFDB, https://www.mgc.ac.cn/VFs/main.htm) and the Comprehensive Antibiotic Resistance Database (CARD, https://card.mcmaster.ca/) [25, 26]. Genome maps were drawn with Proksee (https://proksee.ca/) [27]. The complete phage genome sequence has been deposited in the National Center for Biotechnology Information (NCBI) GenBank database under accession number PX585279.

### Phylogenetic and taxonomic analyses

The complete genome was queried against the NCBI RefSeq viral database and the NCBI nucleotide collection (nt) using blastn (https://blast.ncbi.nlm.nih.gov/; accessed August 2026), with hits covering ≥50% of the query coverage considered close nucleotide-level relatives [28]. Proteome-level neighbours were then selected within the same family-level branch in the Virus Intergenomic Distance Phylogenetic Tree (ViPTree, https://www.genome.jp/viptree) with two near outgroups for context [29]. Pairwise intergenomic similarity and aligned fraction were computed with VIRIDIC (https://rhea.icbm.uni-oldenburg.de/VIRIDIC/) [30]. Phylogeny was inferred with Virus Classification and Tree Building Online Resource (VICTOR, https://ggdc.dsmz.de/victor.php) using the Genome blast Distance Phylogeny (GBDP) approach under recommended settings, based on pairwise amino-acid comparisons; trees were visualized with the *ggtree* package [31, 32].

### Comparative genomic analysis of *V. alginolyticus* isolates

To examine potential bacterial determinants of host specificity, the genomes of the susceptible strain ZS10 and four resistant *V. alginolyticus* isolates, SS6, ZS7, ZS8 and ZS9, were compared. Raw 2×150 bp Illumina paired-end reads were quality-filtered using fastp v1.1.0 and *de novo* assembled using SPAdes v3.13.0 with the --careful option and automatic coverage cutoff. The deposited assemblies were annotated by NCBI using the Prokaryotic Genome Annotation Pipeline [33]. For consistent comparison, the assemblies were reannotated using Prokka v1.13. The annotated genomes were analysed with DefenseFinder (https://defensefinder.mdmlab.fr/) and Kaptive (https://kaptive-web.erc.monash.edu/) to identify putative defence systems and capsular serotypes, while O-antigen and outer-membrane protein candidates were retrieved from the same annotations for comparative assessment [34–36].

### Bactericidal efficacy assay

To evaluate lytic activity of the phage against *V. alginolyticus* ZS10, killing assays were conducted at different MOIs [37]. Early exponential-phase cultures were inoculated into marine broth 2216E at a 1 : 50 ratio and incubated at 28 °C with shaking at 160 r.p.m. until the culture reached OD_600_=0.10, corresponding to ~10^7^ c.f.u. ml^−1^. Cultures were then infected at MOIs of 0.01, 0.1, 1, 10 and 100. The mixtures were dispensed into 96-well microplates (200 µl/well) and incubated quiescently at 28 °C for 24 h in a multimode microplate reader (Synergy H1, BioTek, USA). OD at 600 nm (OD_600_) was recorded every 30 min, with the plate shaken for 5 s before each reading to ensure homogeneity. Each treatment included eight biological replicates, with marine broth 2216E serving as the negative control and bacteria without phage serving as the positive (growth) control.

### Quantitative assessment of phage-mediated biofilm inhibition and disruption

To differentiate the inhibitory effects of the phage during biofilm development from its degradative activity against established biofilms, two experimental setups were designed: an inhibition assay (phage introduced at bacterial inoculation) and a degradation assay (phage applied after biofilm maturation). Total biofilm biomass was quantified through crystal violet staining followed by acetic acid elution, with endpoint measurements recorded as absorbance at OD_595_ [38]. The details of staining and quantification procedures are provided in Text S2.

Briefly, host bacterium *V. alginolyticus* ZS10 was grown overnight in marine broth 2216E at 28 °C with agitation at 180 r.p.m., then adjusted to OD_600_=0.1 (~10^7^ c.f.u. ml^−1^) using fresh marine broth 2216E. Bacterial suspensions were transferred into sterile 24-well plates at 1.0 ml per well and maintained under static conditions at 28 °C to facilitate biofilm development. In the inhibition assay, the phage was introduced concurrently with bacterial inoculation at a final volume of 1.0 ml per well at various MOIs (100, 10, 1, 0.1 and 0.01), with biofilm biomass assessed at 3 and 8 h post-inoculation. Wells receiving *V. alginolyticus* ZS10 alone served as the control. For the degradation assay, biofilms were allowed to mature under static incubation before intervention. Spent medium was carefully removed, and wells underwent gentle washing twice using 1.0 ml sterile PBS. Fresh marine broth 2216E containing the phage was then added (1.0 ml per well) to achieve final infection densities ranging from 10^5^ to 10^9^ p.f.u. ml^−1^, with sampling conducted 3 and 8 h following phage application.

### Phage inhibition of bacterial colonization on *S. hemiphyllum* blade surfaces

An ex vivo colonization assay was designed to evaluate the inhibition of *V. alginolyticus* by the phage on macroalgal surfaces. Blades were cut into 1×1 cm segments and gently rinsed three times with sterile artificial sea water (ASW) to remove loosely attached bacteria. Each experimental well contained one blade segment in a final volume of 1.0 ml, consisting of 800 µl of ASW, 100 µl of *V. alginolyticus* ZS10 suspension and 100 µl of phage. The *Vibrio* inoculum was adjusted to a final concentration of 10^6^ c.f.u. ml^−1^, and the phage was added to achieve MOIs of 100, 1 and 0.01, respectively. Control wells were prepared identically but without phage. The plates were statically incubated at 28 °C, and 100 µl of the supernatant was collected at 3 and 8 h post-inoculation for planktonic bacterial enumeration. After removing the remaining liquid and gently washing the blades twice with 1.0 ml ASW to eliminate loosely attached cells, the segments were then transferred to centrifuge tubes containing 1.0 ml sterile artificial seawater and vortexed for 2 min to detach surface-associated bacteria. Both planktonic and surface-detached samples were serially diluted and plated onto marine agar 2216E, followed by overnight incubation at 28 °C for colony enumeration. All experiments were performed in triplicate.

### Statistical analysis

All data were quantified as the mean±sd. One-way ANOVA and Tukey’s multiple range test for this study were performed using SPSS statistical package version 26.0 (SPSS, Inc., Chicago, IL, USA) and Origin Pro 2022 (https://www.originlab.com/). Differences were considered significant at *P*<0.05.

## Results

### Morphology and infection dynamics of phage R39Z

The lytic phage infecting *V. alginolyticus* ZS10 was isolated from the surface of *S. hemiphyllum* and designated vB_ValP-R39Z (abbreviated as R39Z). In the double agar overlay assay, phage R39Z produced circular, clear plaques with sharp margins and no halo after 5 h of incubation, which, by 24 h, had enlarged and developed a distinct peripheral halo, with the average plaque diameter (including halo) increased from 0.54±0.40 mm at 5 h to 2.52±0.61 mm at 24 h, corresponding to an expansion rate of ~0.10 mm h^−1^ (Fig. 2a). TEM analysis revealed that R39Z belongs to the podovirus morphotype, characterized by an icosahedral head measuring 87.79±9.22 nm in diameter and a short, non-contractile tail 17.87±5.86 nm in length and 10.51±3.44 nm in width (*n*=12) (Fig. 2b). Chloroform sensitivity testing showed no significant difference between the control and the chloroform-treated group (*P*>0.05), indicating that R39Z is resistant to chloroform, consistent with a non-enveloped virion (Fig. 2c).

**Fig. 2.**
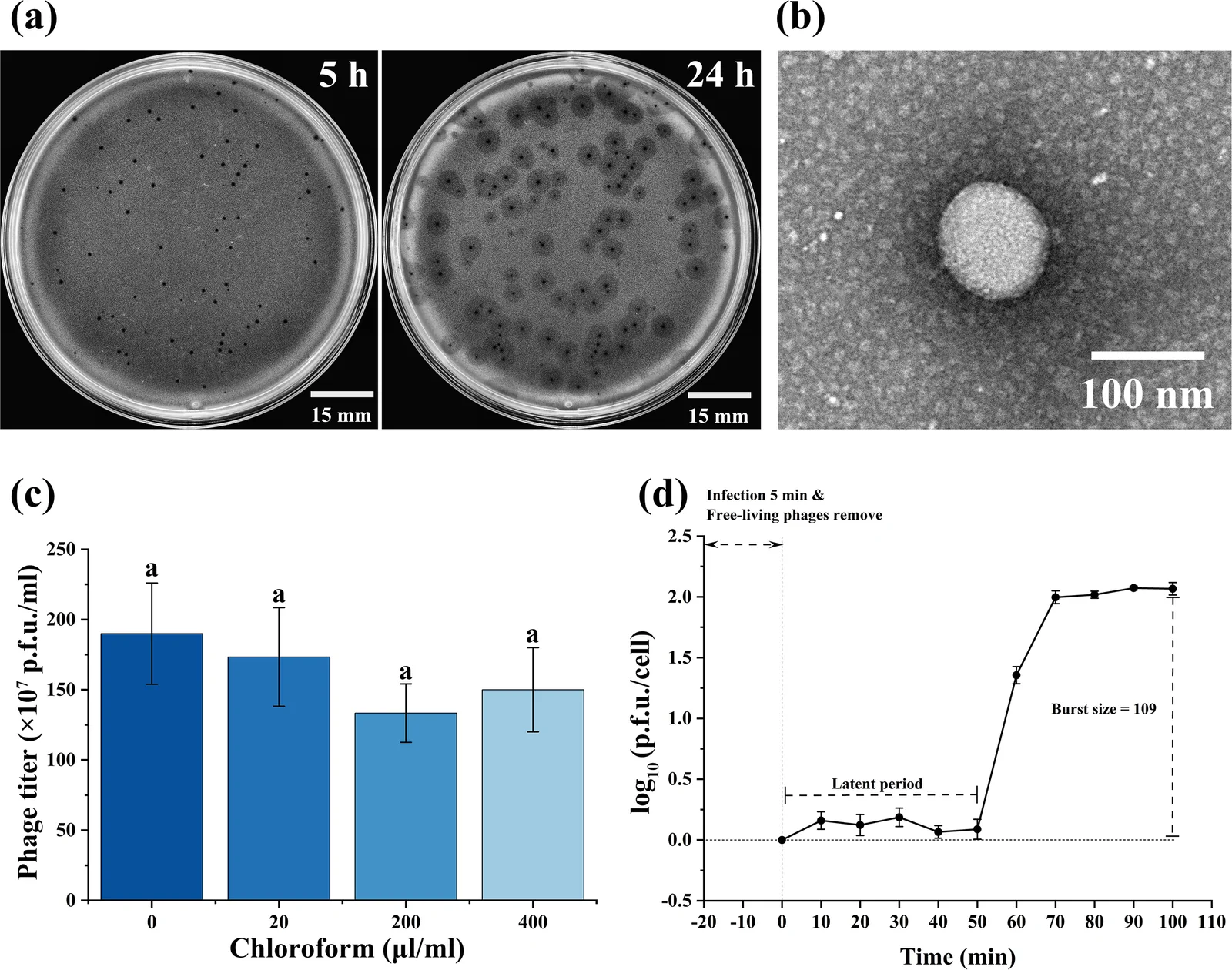
Morphology and infection dynamics of phage R39Z. (**a**) Plaque morphology of phage R39Z on *V. alginolyticus* ZS10 after 5 and 24 h of incubation in the double agar overlay assay. (**b**) Transmission electron micrograph of phage R39Z, showing a short non-contractile tail. (**c**) Chloroform sensitivity assay comparing phage titres between untreated and chloroform-treated samples. Data represent mean±sd of three replicates, and different letters indicate significant differences (*P*<0.05). (**d**) One-step growth curve of phage R39Z infecting *V. alginolyticus* ZS10 (mean±sd, *n*=3).

A one-step growth analysis resolved three phases of the infection cycle, namely the latent period, burst period and plateau period (Fig. 2d). The latent period was ~50 min long, and no detectable increase in phage titre was observed, reflecting the intracellular replication stage preceding host lysis. A rapid rise in phage abundance could be observed thereafter, eventually reaching a plateau at around 70 min. Consequently, the average burst size was 109 p.f.u. per infected cell (p.f.u./cell), indicating efficient progeny production per infection cycle. Such a high burst yield suggests that the relatively long latent period of R39Z allows sufficient time for extensive replication and assembly of progeny virions, reflecting its strong replicative capacity and efficient utilization of host resources.

### Host range

Phage R39Z exhibited a strictly strain-specific host range, infecting only *V. alginolyticus* ZS10, with no detectable lytic activity against other tested strains (Fig. 3). Notably, the tested strains included 20 bacterial isolates from the *S. hemiphyllum* cultivation environment, 11 of which belonged to the genus *Vibrio*. Among these, four isolates were identified as *V. alginolyticus* sharing close phylogenetic relationships with *V. alginolyticus* ZS10, yet none of them were sensitive to R39Z, underscoring an extremely narrow phage–host specificity. Further assessment of lytic activity demonstrated that phage R39Z could efficiently lyse its original host, *V. alginolyticus* ZS10, even at a low concentration of 10³ p.f.u. ml^−1^ (Fig. S1). Therefore, phage R39Z displayed extremely narrow host specificity, exclusively infecting its original host *V. alginolyticus* ZS10 while efficiently lysing it even at low phage concentrations.

**Fig. 3.**
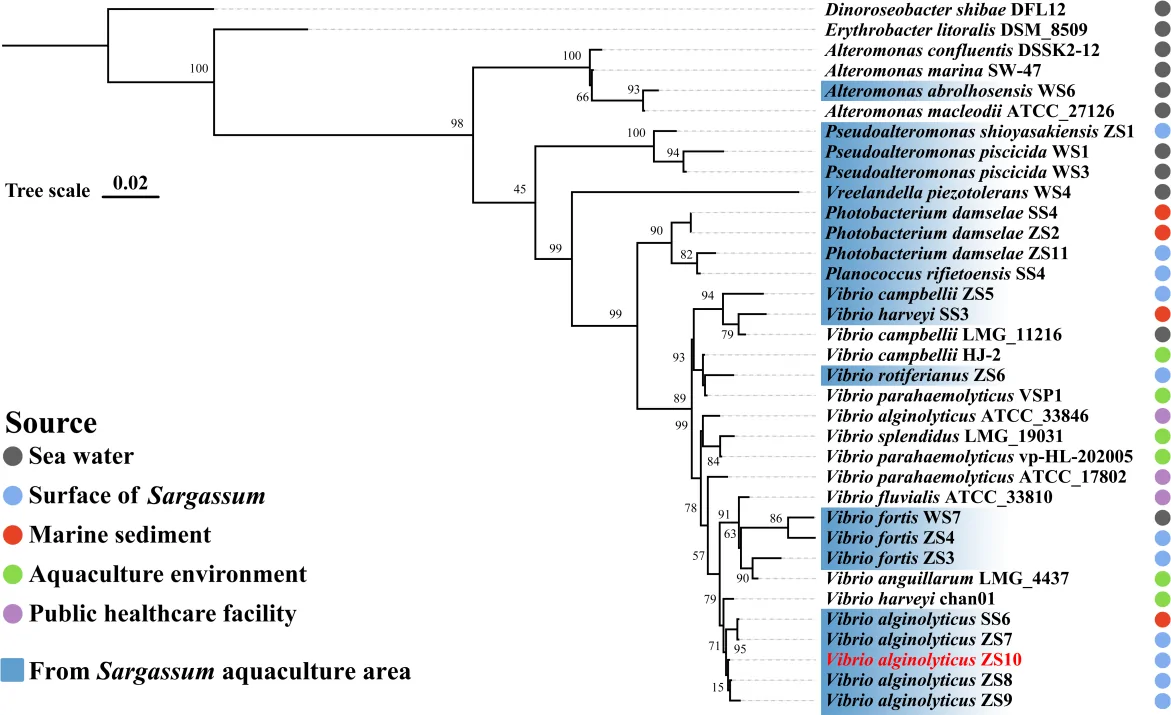
Phylogenetic tree illustrating the host range of phage R39Z. The tree was constructed based on 16S rRNA gene sequences of *Vibrio* and non-*Vibrio* strains tested in this study. Strain susceptible to R39Z infection is highlighted in red, while resistant strains are shown in black.

### Environmental stabilities of phage R39Z

The titres of phage R39Z were measured across pH 2–12 and temperatures of 4–65 °C at 3, 24 and 48 h (Fig. 4). Within 3 h, R39Z retained >60% activity across a broad pH range (pH 5–10), while extreme pH values (pH 2 or 12) rapidly abolished activity. After 48 h, R39Z remained stable at pH=7–9, retaining 79–89% of initial titres, while survival declined to 46–65% at pH=5–6, 25% at pH=4 and 18% at pH=11, and no plaques were observed after 3 h at pH=2 or pH=12. Thermal stability tests showed that R39Z remained stable within 4–35 °C, maintaining survival rates above 75% over 48 h, whereas titres decreased rapidly at higher temperatures, falling below detection after 24 h at 45 °C and becoming completely inactivated within 3 h at 55–65 °C. Overall, these results indicate a stability range of pH=7–9 and temperatures of 4–35 °C for R39Z, outside of which survival declined rapidly.

**Fig. 4.**
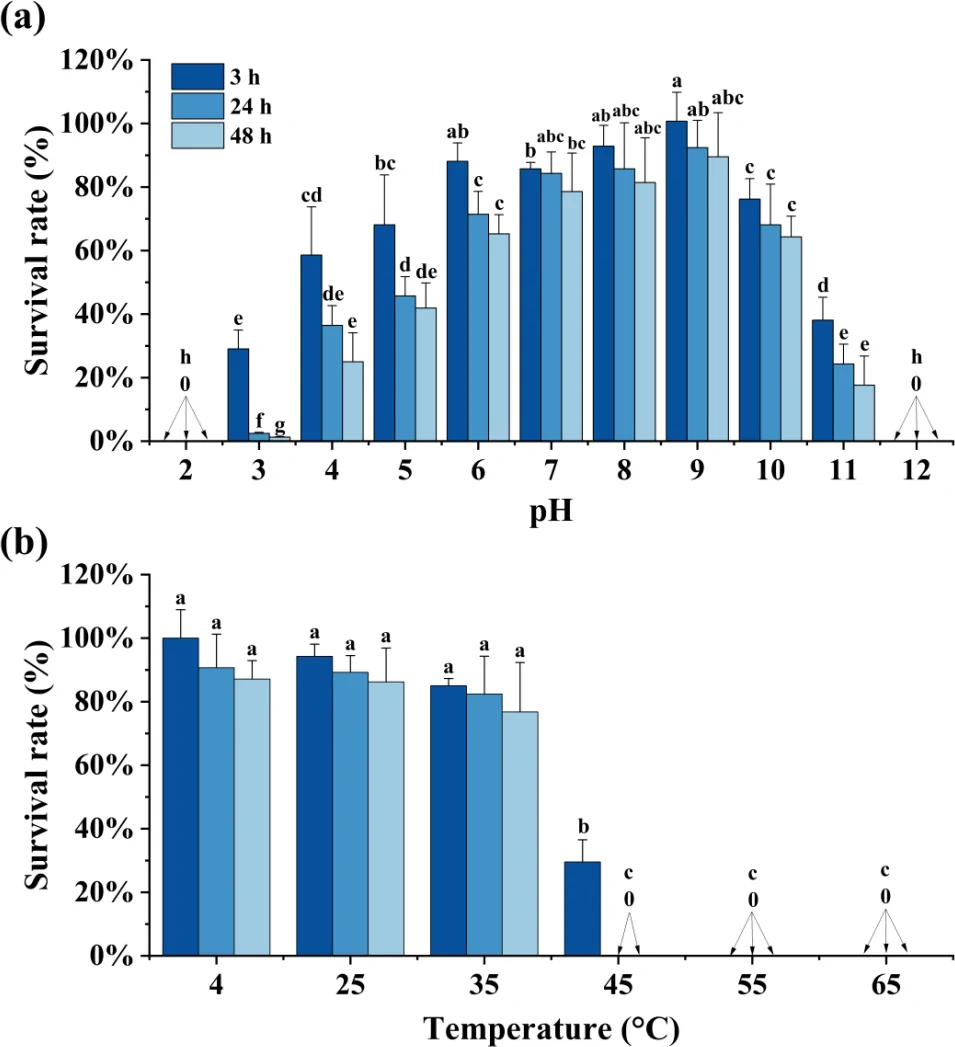
Environmental stability of R39Z. (**a**) pH stability of R39Z assessed after 3–48 h incubation at pH 2–12. (**b**) Thermal stability of R39Z assessed after 3–48 h incubation at 4–65 °C. Data represent mean±sd of three replicates, and different letters indicate significantly different groups (*P*<0.05).

### Genomic characteristics

PhageTerm analysis revealed no fixed genomic termini, and the distributions of read coverage and read starts were consistent with headful (pac) DNA packaging. The genome of phage R39Z is dsDNA, 71,824 bp in length, with a G+C content of 37.73 mol%. Genome annotation identified 83 ORFs, 33 of which were assigned putative functions spanning eight categories: DNA, RNA and nucleotide metabolism (11), tail (6), head and packaging (5), lysis (3), connector (1), transcription regulation (2), AMG (2) and others (3) (Fig. 5a; Table S3). Notably, two AMGs were concordantly annotated by PHROGs and DRAM-v, with one encoding a putative R67-family dihydrofolate reductase for which AlphaFold-Multimer predicted a homotetramer featuring a single central pore at the subunit interface, a configuration characteristic of plasmid-encoded R67 dihydrofolate reductases (Fig. 5b). The other encodes a thymidylate synthase predicted to form a homodimer with the catalytic site at the dimer interface, retaining the conserved catalytic cysteine typical of folate-dependent thymidylate synthases (Fig. 5c). Three tRNA genes were predicted, corresponding to Asn (anticodon GTT), Gln (TTG) and Trp (CCA). Additionally, no genes associated with antibiotic resistance, virulence, integrase or canonical repressors were detected by CARD or VFDB.

**Fig. 5.**
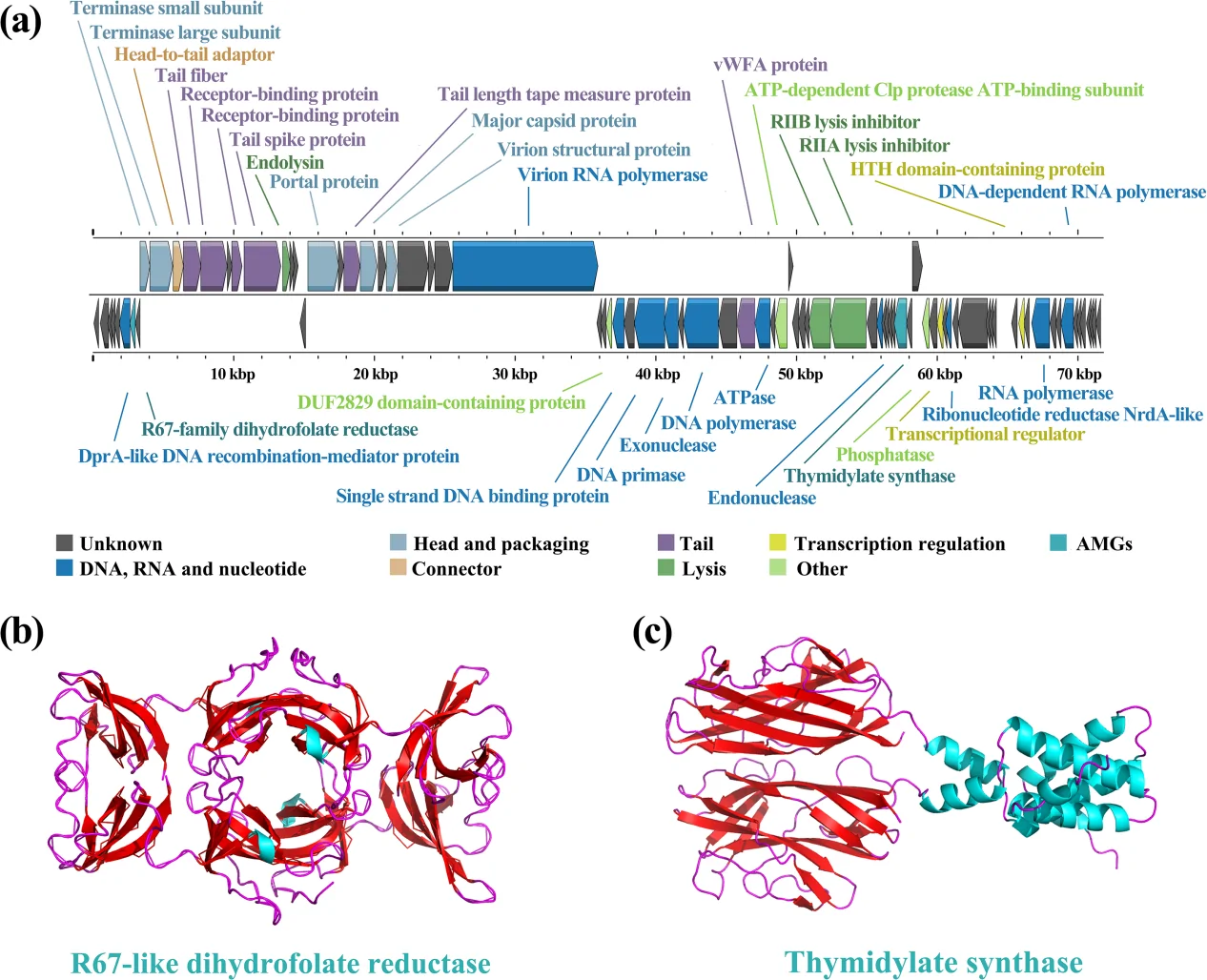
Linear genome map of phage R39Z and predicted structures of proteins encoded by AMGs. (**a**) Genome map of R39Z generated using Proksee. Genes encoded on the forward (+) strand are shown above the central axis, and genes encoded on the reverse (−) strand are shown below; arrows are colour-coded by functional category. Key genes are labelled to delineate the major functional modules of the genome. (b, c) AlphaFold-predicted protein structures visualized in PyMOL: (**b**) putative R67-family dihydrofolate reductase homotetramer with a central catalytic pore; (**c**) thymidylate synthase homodimer with the catalytic site at the dimer interface.

### Phylogeny and taxonomy of phage R39Z

BLASTn analysis against the NCBI RefSeq viral database identified no close nucleotide-level relatives of R39Z, indicating its substantial genetic novelty. The closest match identified in the NCBI nucleotide collection (nt) was bacteriophage ctLUJ1 (MN582077.1), which aligned to only 26.56% of the R39Z genome. To further elucidate its evolutionary position, a ViPTree proteomic analysis based on genome-wide tBLASTx was performed. The resulting tree placed R39Z within the family *Schitoviridae*, where it clustered most closely with bacteriophage ctLUJ1 (Fig. 6a). This close phylogenomic relationship was further supported by the VICTOR/GBDP balanced minimum-evolution tree, in which R39Z and ctLUJ1 formed a well-supported sister group with a bootstrap support of 100% (Fig. 6b). However, despite their close clustering in phylogenomic analyses, VIRIDIC analysis revealed limited genomic relatedness between R39Z and ctLUJ1, with only 19.9% intergenomic similarity, while similarities with the remaining reference phages ranged from 2.2 to 3.9% (Fig. 6c). According to the default VIRIDIC thresholds for species (95%) and genus (70%) demarcation, all reference phages shared less than 70% intergenomic similarity with R39Z. Collectively, these results indicate that R39Z represents a distinct genomic lineage within *Schitoviridae* and suggest that it may represent a novel unclassified genus.

**Fig. 6.**
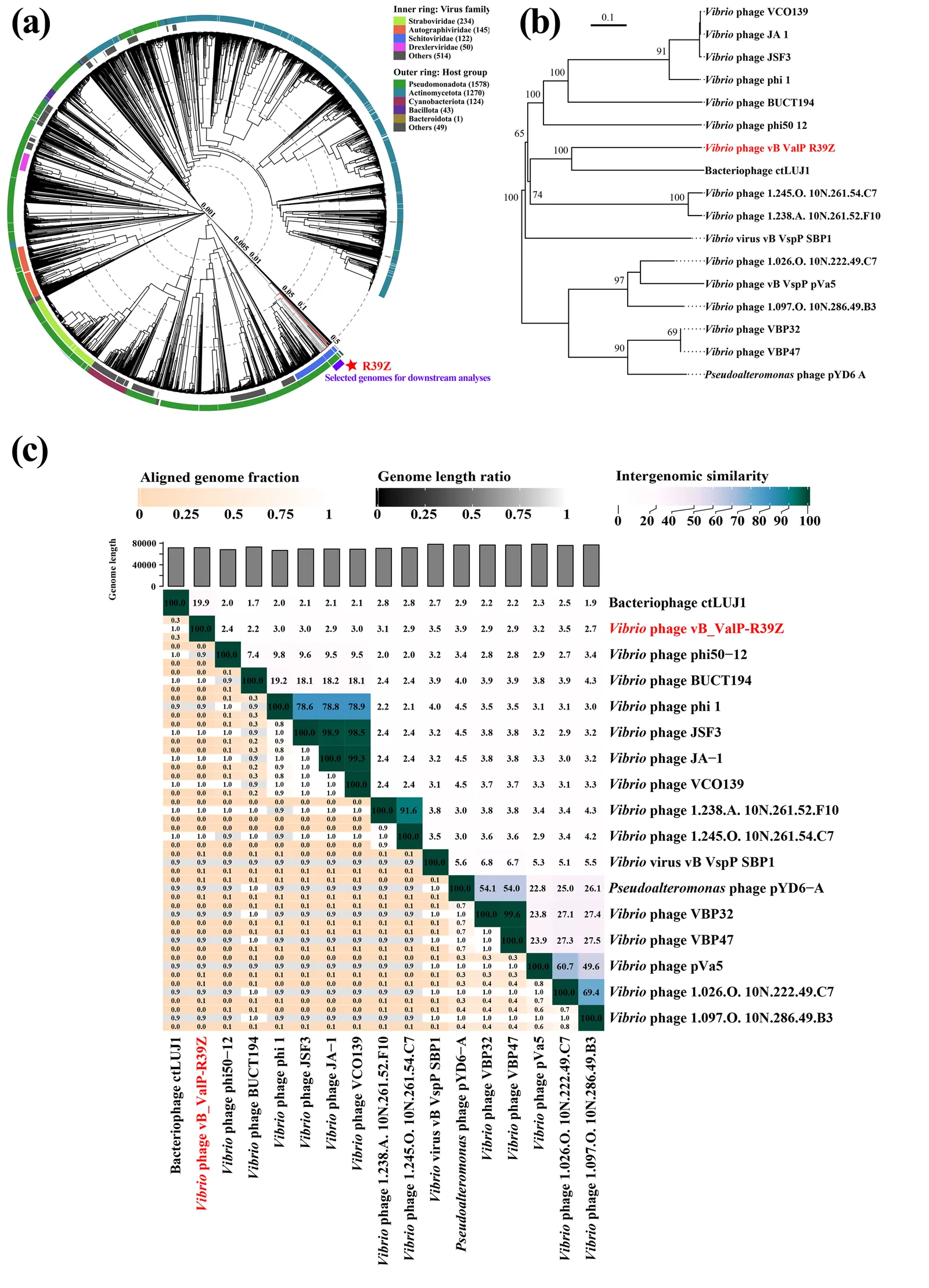
Taxonomic and phylogenetic analysis of R39Z. (**a**) ViPTree proteomic tree based on genome-wide tblastx similarities. (**b**) GBDP tree based on complete or partial genomes of compared phages by the web tool, VICTOR. (**c**) Pairwise intergenomic distances/similarities among viral genomes for 17 phages as per the Virus Intergenomic Distance Calculator.

### Lytic activity and biofilm interference of phage R39Z

Planktonic killing kinetics and the phage’s ability to inhibit biofilm formation and disrupt established biofilms were evaluated to characterize phenotypic traits relevant to the macroalgal surface niche. Killing kinetics at different MOIs (0.01, 0.1, 1, 10 and 100) further demonstrated clear MOI- and time-dependent lysis (Fig. 7a, b). In the uninfected control (*Vibrio* only), OD_600_ increased from 0.24±0.01 at 0 h to 1.34±0.01 at 24 h, whereas R39Z infection markedly suppressed *Vibrio* growth. After 3 h of infection, bactericidal efficiencies reached 74% at an MOI of 100 and decreased progressively with lower MOIs, dropping to 20% at an MOI of 0.01. By 8 h, maximal efficiencies shifted to intermediate MOIs (85% at MOI of 10 and 83% at MOI of 1), whereas the highest MOI (100) showed a lower value of 68%. However, after 24 h, bactericidal efficiency increased as MOI decreased, with suppression at MOI of 100 dropping to 6% while the lowest MOI (0.01) maintained 42%, possibly because higher MOIs promoted the emergence of more phage-resistant bacteria. These results indicate that high MOIs achieve rapid, short-term suppression of *Vibrio* growth, whereas low MOIs sustain longer-term inhibitory effects.

**Fig. 7.**
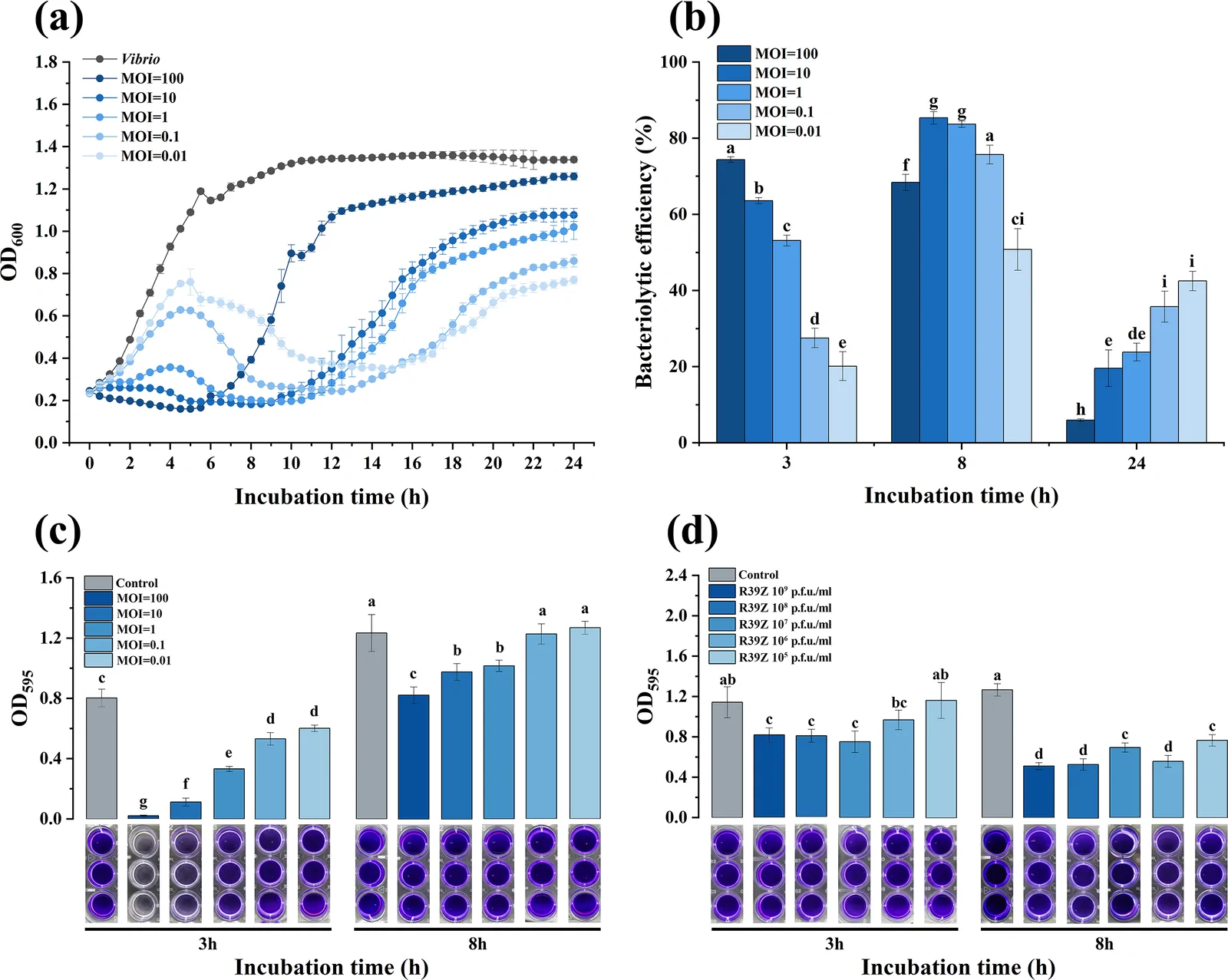
Lytic activity and biofilm inhibition/disruption capacity of phage R39Z. (**a**) Bacterial lysis curves across different MOIs (0.01, 0.1, 1, 10 and 100), with *Vibrio* without phage serving as the control (mean±sd, *n*=8). (**b**) Bactericidal efficiencies at 3, 8 and 24 h post-infection at different MOIs (mean±sd, *n*=8). Different letters represent significant differences (*P*<0.05). (**c**) Biofilm inhibition by R39Z under co-inoculation at different MOIs at 3 and 8 h (mean±sd, *n*=3). (**d**) Biofilm disruption of pre-formed mature biofilms by R39Z (10^5^–10^9^ p.f.u. ml^−1^) at 3 and 8 h after R39Z treatment (mean±sd, *n*=3). Different letters indicate significant differences within the same time point (*P*<0.05).

To link infection kinetics to an ecologically relevant attached lifestyle, we quantified the effects of R39Z on biofilm formation and on pre-established biofilms using a crystal violet microplate assay (Fig. 7c, d). In the inhibition assay, the control OD_595_ was 0.80±0.06 at 3 h and 1.23±0.12 at 8 h. At 3 h, R39Z reduced biofilm biomass by 25–98% compared to the control across a wide range of MOIs (0.01–100). At 8 h, significant inhibition was observed at MOIs of 1–100 (17–33% reduction, *P*<0.05), while low MOI (0.01–0.1) groups showed no significant decrease compared to controls (*P*>0.05). Overall, higher MOIs exhibited stronger biofilm formation inhibitory activity in the inhibition assay. In the disruption assay, the control OD_595_ was 1.14±0.15 at 3 h and 1.27±0.06 at 8 h. At 3 h, R39Z significantly reduced pre-formed biofilm biomass by 28–34% at 10^7^–10^9^ p.f.u. ml^−1^ (*P*<0.05), while no significant reduction was observed at 10^5^–10^6^ p.f.u. ml^−1^ (*P*>0.05). At 8 h, R39Z reduced pre-formed biofilm by 39–59% across 10^5^–10^9^ p.f.u. ml^−1^, compared to the control group. Taken together, these results demonstrate that R39Z is capable of both inhibiting *de novo* biofilm formation and disrupting pre-formed biofilms in a concentration- and time-dependent manner, supporting its potential role in interfering with the *Vibrio* attached lifestyle on macroalgal surfaces.

### Inhibition of *V. alginolyticus* by phage R39Z on *Sargassum* surfaces

The efficiency of phage R39Z in inhibiting *V. alginolyticus* colonization on macroalgal surfaces was evaluated using ex vivo *S. hemiphyllum* blades. Before application, we ensured that there was no background *Vibrio* contamination on the natural *Sargassum* blades that could affect the test results. The results showed that the number of *V. alginolyticus* ZS10 in the control group increased continuously over 8 h in both the surrounding seawater and on the blade surfaces, while the phage treatment groups showed significant inhibition (*P*<0.05) (Fig. 8). In the planktonic fraction, phage treatment at MOIs of 100 and 1 could significantly reduce the planktonic bacterial count by more than 99% at 3 h, while low-MOI treatment (MOI=0.01) caused an ~91% reduction relative to the control (*P*<0.05) (Fig. 8b). By 8 h, the low-MOI treatment (MOI=0.01) also exhibited a significant bactericidal effect, reducing the planktonic bacteria by more than 99%. Similarly, phage treatment significantly inhibited bacterial colonization on the blade surfaces. At 3 h, phage treatment reduced surface-associated cells by more than 99% across MOIs of 0.01–100 (*P*<0.01) (Fig. 8c). At 8 h, phage treatment at different MOIs reduced the abundance of surface-associated *V. alginolyticus* ZS10 by ~99%. In conclusion, phage R39Z effectively reduced the abundance of *V. alginolyticus* ZS10 both on the surface of *Sargassum* blades and in the surrounding water.

**Fig. 8.**
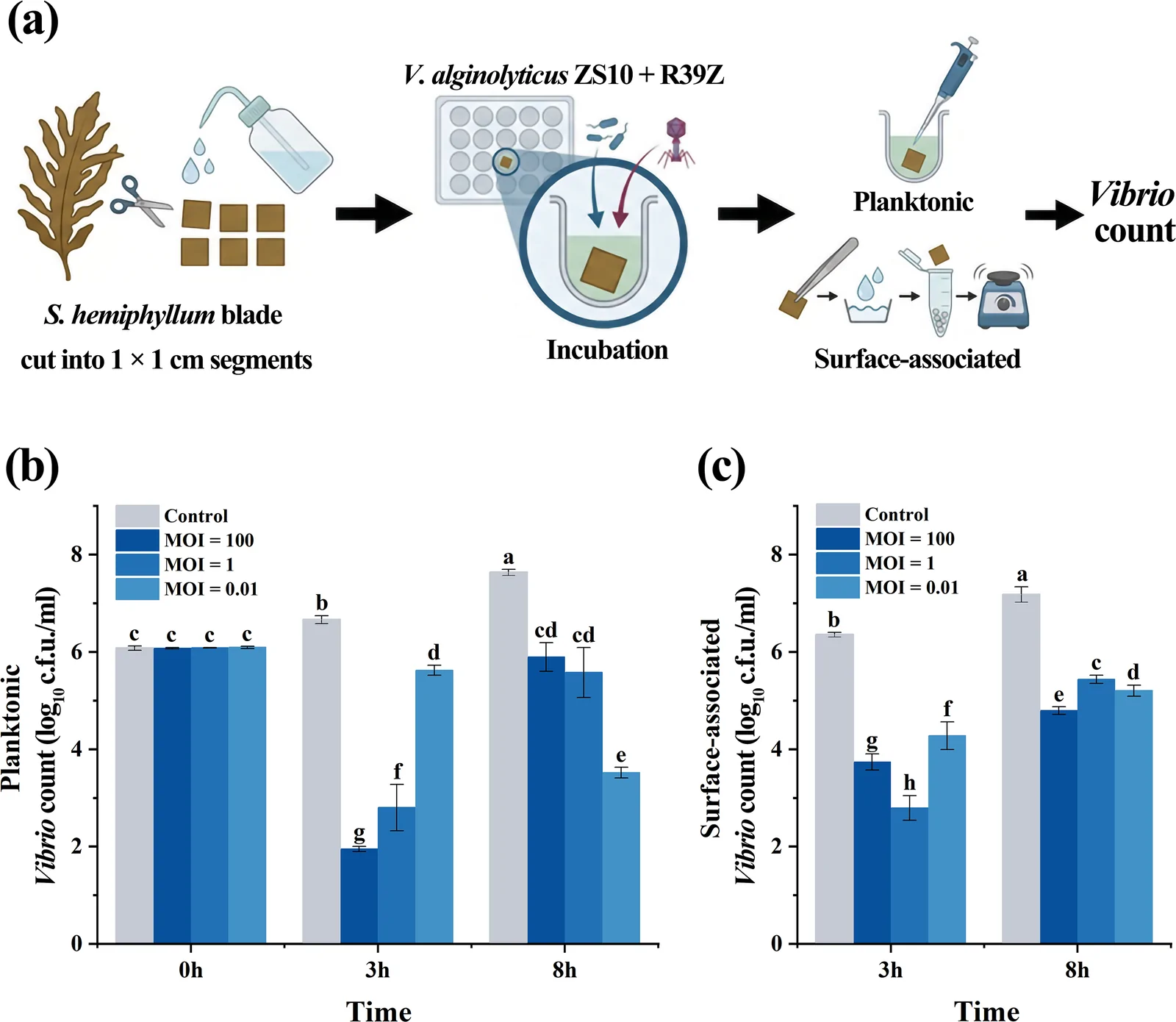
Inhibitory effect of phage R39Z on *V. alginolyticus* ZS10 in an ex vivo *S. hemiphyllum* blade assay. (**a**) Workflow of the ex vivo *S. hemiphyllum* assay. (**b**) Viable counts of planktonic *V. alginolyticus* ZS10 in the surrounding medium at 0, 3 and 8 h following phage treatment at MOIs of 100, 1 and 0.01. (**c**) Viable counts of surface-associated *V. alginolyticus* ZS10 recovered from *S. hemiphyllum* blade surfaces at 3 and 8 h under the same treatments. Bars represent mean±sd (*n*=3). Different letters indicate significant differences within the same time point (*P*<0.05).

## Discussion

Within macroalgal microbial consortia, *V. alginolyticus* exhibits ecological flexibility, shifting between mutualism and pathogenicity depending on environmental conditions, which underscores the role of phage-mediated regulation in maintaining microbial balance within the holobiont. In this context, the phage R39Z, isolated from the surface of *S. hemiphyllum* and characterized in this study, offers an ecologically meaningful tool for exploring the tripartite interactions among macroalgae, *Vibrio* and their phages.

The infection dynamics and environmental resilience of R39Z offer insights into its replication strategy and ecological fitness within this niche. The one-step growth curve revealed a latent period of ~50 min, followed by the onset of the plateau phase around 70 min, with an average burst size of about 109 p.f.u./cell. Reported burst sizes of vibriophages generally range from 2 to 180 p.f.u./cell, placing R39Z at the upper end of this range and indicating a relatively high replication efficiency per infection cycle [39]. This phenotype is likely supported by a nucleotide metabolism module encoded by R39Z, including thymidylate synthase (ThyA; ORF57), a putative R67-family dihydrofolate reductase (R67-family DHFR; ORF7) and ribonucleotide reductase (RNR; ORF64), which together promote the *in situ* synthesis of dTMP and dNTPs [40, 41]. ThyA and the putative R67-family DHFR may therefore help buffer phage nucleotide synthesis against fluctuations in host metabolic activity, potentially supporting R39Z replication under the variable conditions of the macroalgal phycosphere. Furthermore, the R39Z-encoded R67-family DHFR may sustain nucleotide synthesis under trimethoprim pressure, thereby potentially altering phage–bacterium interactions, although this remains to be tested [42]. Moreover, R39Z encodes a virion-associated RNA polymerase, which may facilitate early transcription with reduced dependence on host transcriptional machinery [43]. Together, these features suggest a mechanistic basis for the relatively rapid replication and high infection efficiency of R39Z. However, rapid replication alone is insufficient to ensure ecological relevance in the macroalgal phycosphere, as sustained influence also depends on whether the phage remains infective under the physicochemical conditions of this niche. Environmental stability assays determined that R39Z retained high infectivity within a pH range of 7–9 and temperatures from 4 to 35 °C, while rapid inactivation occurred under highly acidic, alkaline or elevated temperature conditions. Given that *Sargassum* inhabits warm-temperate to tropical waters, where microenvironmental pH can reach up to 9 and temperatures typically range from 18 to 30 °C, these stability characteristics suggest that R39Z is well adapted to the physicochemical conditions of the macroalgal surface habitat [44].

However, ecological fitness alone does not determine ecological impact. The ability of R39Z to regulate macroalgal-associated *Vibrio* populations also depends on the breadth and determinants of its host range. Within the macroalgal phycosphere, R39Z exhibited an extremely narrow host range, lysing only *V. alginolyticus* ZS10. Given that phage host range is primarily determined by tail-associated receptor-binding proteins during the adsorption phase and may be further restricted by intracellular anti-phage defence systems, this study investigated both phage-encoded host-recognition modules and bacterial determinants of susceptibility [45, 46]. In the *V. cholerae* phage ICP2, mutations in the putative tail fibre Gp25 altered recognition of the OmpU receptor, while additional mutations in the putative adhesin Gp23 enhanced infection of receptor variants, providing experimental evidence that tail-associated proteins can modify host specificity [47]. The R39Z genome encoded multiple tail-associated structural proteins, including ORF13, ORF15 and ORF16, which may participate in host recognition. However, phage-encoded adsorption modules alone could not explain why only *V. alginolyticus* ZS10 was permissive. To further identify host-side determinants of susceptibility, we compared receptor-related loci and intracellular anti-phage defence repertoires among five closely related *V. alginolyticus* strains isolated from the *Sargassum* cultivation environment (Fig. S2). Comparative analysis indicated that neither the largely absent classical O-antigen biosynthesis systems nor the highly conserved outer-membrane porin profiles could account for the observed infection outcomes [48, 49]. By contrast, these closely related strains exhibited highly variable capsular polysaccharide biosynthesis architectures, along with completely distinct and combinatorial repertoires of intracellular defence islands, such as BREX, Abi-like and Lamassu modules [50, 51]. Such variation is mechanistically important, as surface polysaccharides can influence phage adsorption either by serving as direct receptors or by masking underlying receptor structures, while intracellular defence systems act predominantly after adsorption to restrict phage replication. The comparative patterns indicate that resistance is not determined by the total number of defence systems but by their specific combinations [52]. Therefore, the narrow host range of R39Z likely reflects the combined effects of fine-scale differences in surface polysaccharides and strain-specific intracellular defence repertoires, which is consistent with the bacterial ‘pan-immune system’ framework [53]. From an ecological perspective, these results suggest that the role of R39Z is not to broadly eliminate *Vibrio* strains colonizing the macroalgal surface, but rather to selectively suppress the host strain.

The observed planktonic infection dynamics and host-specific interactions of R39Z suggest broader ecological implications, motivating an examination of its impact on biofilm formation and persistence in surface-associated *Vibrio* populations. The biofilm biomass data extend these ecological inferences from planktonic killing to the sessile state, which is particularly relevant in the macroalgal phycosphere. In this environment, *Vibrio* populations, including *V. alginolyticus*, tend to be surface-associated, and biofilms can enhance persistence while, under certain conditions, facilitating pathogenicity [54]. When R39Z was co-inoculated with bacteria, crystal violet–quantified *de novo* biofilm biomass (a proxy for total biomass rather than viability) declined substantially across a broad MOI range at early time points. By 8 h, however, the strongest inhibition had shifted towards higher MOIs, a pattern consistent with rapid initial lysis that becomes attenuated at lower phage inputs as biofilms consolidate. In assays targeting pre-formed biofilms, R39Z still reduced established biomass, though this required either higher phage concentrations or extended exposure times, suggesting that biofilm architecture itself may impose kinetic barriers that limit phage access to matrix-embedded cells [55]. From an ecological perspective, these results suggest that R39Z may function more as a suppressor of susceptible *Vibrio* populations than as an agent of complete host elimination. Such sustained but incomplete suppression could be advantageous in the macroalgal phycosphere, where continued host availability may support persistent phage propagation [56]. Taken together with R39Z’s environmental stability and infection kinetics, these findings suggest that the phage plays a crucial role in modulating its host across both planktonic and biofilm-associated states.

To bridge the gap between *in vitro* biofilm experiments and natural habitats, our ex vivo *Sargassum* system provides a more ecologically relevant context for understanding how this phage-mediated regulation operates within the macroalgal phycosphere. Viable counts clearly indicated that R39Z effectively reduced active *Vibrio* populations in both the planktonic fraction and on macroalgal surfaces. Notably, the anti-colonization effect on the macroalgal surface was slightly weaker than the rapid clearance observed in the planktonic fraction. This difference suggests that the EPS-rich macroalgal surface may provide a spatial refuge and partial kinetic barrier that reduces phage diffusion or access to embedded host cells [55]. Despite these potential physical constraints, R39Z still reduced surface *Vibrio* by ~99%, highlighting its crucial role as a natural ecological regulator – effectively preventing the massive colonization of pathogens while maintaining microbial balance within the macroalgal symbiotic community.

Although the lytic activity demonstrated by R39Z highlights its potential as a sustainable biological control agent for macroalgae aquaculture, its application must be considered within the context of phage–host co-evolution dynamics. Unlike broad-spectrum antibiotics, R39Z exhibits a narrow host range, an ecological feature that allows precise targeted removal of specific pathogenic *Vibrio* strains without disrupting the commensal microbiome essential for macroalgal health. Our genomic analysis of diverse *Vibrio* isolates revealed that these co-existing *Vibrio* strains harbour diverse capsule structures and variable combinations of intracellular defence systems. This genomic heterogeneity likely underlies the inability of R39Z to infect the broader *Vibrio* community. Therefore, the application of a single phage such as R39Z in open aquaculture systems may be constrained by its narrow host range and the intrinsic resistance of diverse bacterial populations. Future biocontrol strategies in coastal aquaculture may therefore benefit from phage cocktails informed by bacterial defence-system profiling. Such approaches may help broaden strain coverage and improve the robustness of phage-based management of pathogenic *Vibrio* in environmentally complex systems. Beyond host range considerations, the genetic safety of phage application also warrants attention. Although no recognizable antimicrobial resistance or virulence genes were identified in the R39Z genome, its pac-type packaging strategy allows for mispackaging of host DNA, a prerequisite for generalized transduction [57]. This process was not tested experimentally, yet its ecological implications for phage-mediated gene flow within macroalgal-associated microbial communities offer an additional perspective.

## Conclusion

In summary, phage R39Z represents a previously uncharacterized *Vibrio*-infecting phage, isolated from the surface of the brown alga *S. hemiphyllum*. Through comprehensive characterization of its infection dynamics, environmental stability, host specificity and genomic architecture, R39Z was established as a novel genus-level lineage. Biofilm assays and an ex vivo *Sargassum* colonization approach further demonstrated that R39Z effectively inhibits the growth of *V. alginolyticus* ZS10 in planktonic and surface-associated states, reducing the c.f.u. counts of attached *Vibrio* by ~99%. Collectively, this study provides an ecologically relevant framework for understanding phage–host interactions on macroalgal surfaces. These findings also support future exploration of phage-based biocontrol strategies, including defence-system-informed phage cocktails, for managing pathogenic *Vibrio* in coastal aquaculture.

## Supplementary material

10.1099/mgen.0.001873 Supplementary Material 1.

10.1099/mgen.0.001873 Table S1.

## Abbreviations

AMGs: auxiliary metabolic genes
ASW: artificial sea water
CARD: Comprehensive Antibiotic Resistance Database
DHFR: dihydrofolate reductase
EPSs: extracellular polymeric substances
GBDP: Genome blast Distance Phylogeny
MOIs: multiplicities of infection
OD_600_: Optical density at 600 nm
ORFs: open reading frames
p.f.u./cell: plaque-forming units per infected cell
TEM: transmission electron microscopy
VFDB: Virulence Factors Database
VICTOR: Virus Classification and Tree Building Online Resource
ViPTree: Virus Intergenomic Distance Phylogenetic Tree
