# Supplementary material for: A Sargassum-associated phage slows Vibrio alginolyticus persistence on macroalgal surfaces
Source: Microb Genom. 2026 Nov 10;12(11):001873. doi: 10.1099/mgen.0.001873 (PMC13749740; doi:10.1099/mgen.0.001873)
Supplement: Supplementary file 1 [file mgen-12-01873-s001.pdf]

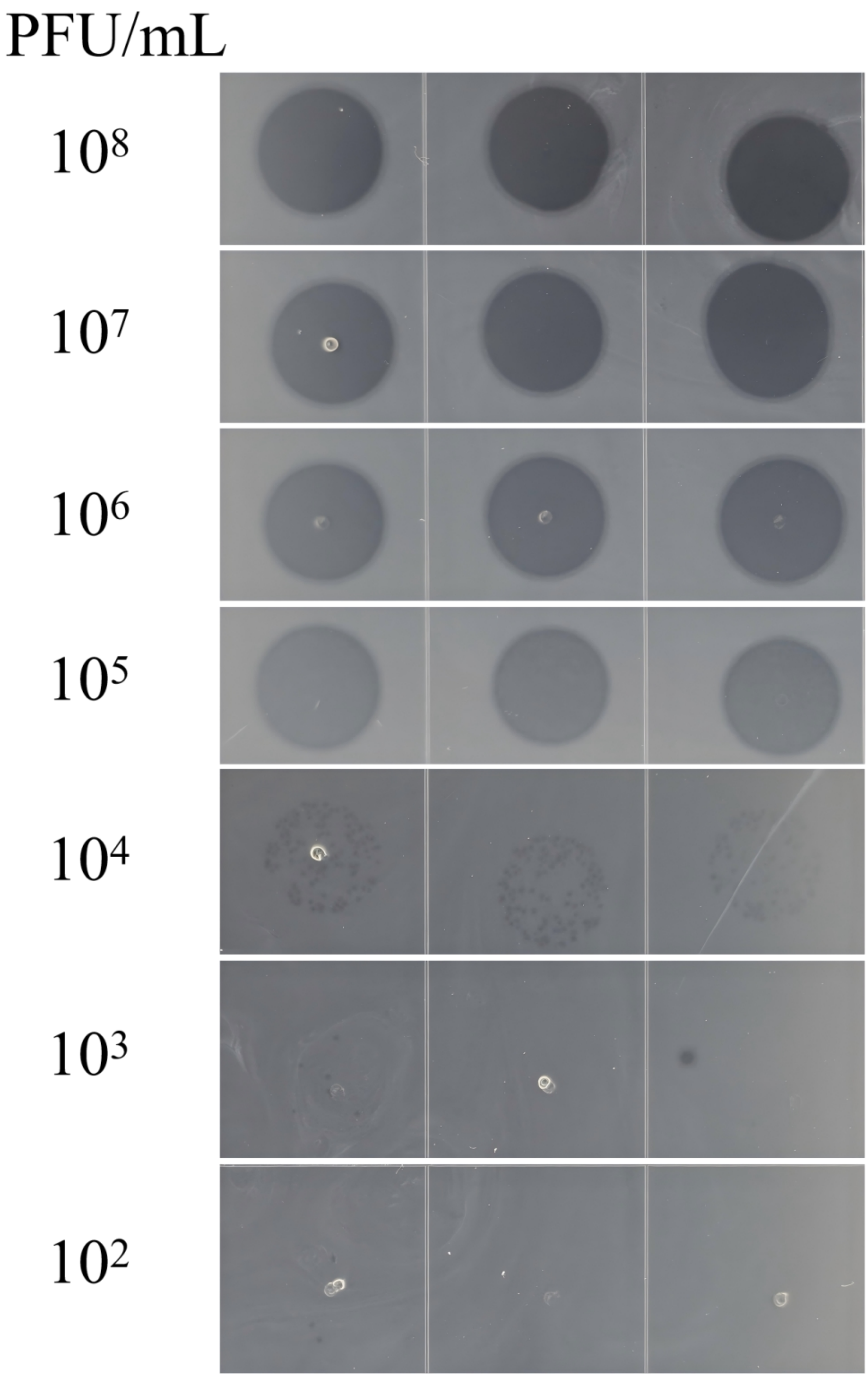


**Supplementary figure 1.** Spot assay images showing titration images from $10^2$ to $10^8$ PFU/mL.

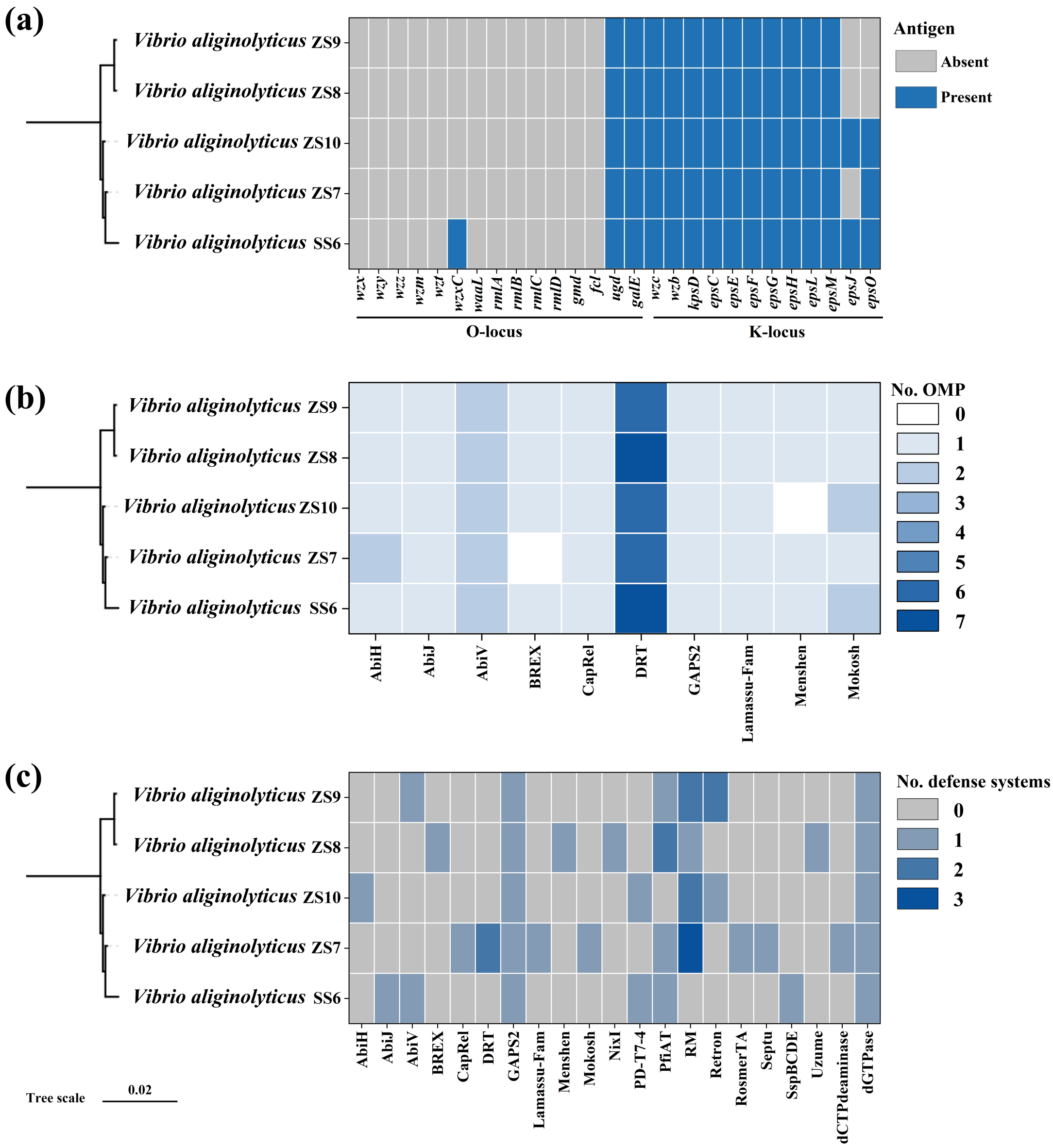


**Supplementary figure 2.** Comparative heatmaps illustrating the distribution of surface structure genes and intracellular defense mechanisms across five *Vibrio alginolyticus* strains. (a) Presence/absence heatmap of O- and K-locus genes (blue, present; grey, absent), summarizing biosynthetic modules for O-antigen and capsular/EPS formation. (b) Copy-number variation of representative outer membrane protein (OMP) families. (c) Predicted anti-phage defense systems identified using DefenseFinder.

**Supplementary Text 1. Details of the MOIs analysis.**

To determine the optimal multiplicity of infection (MOI), serial MOI gradients ranging from 0.0001 to 100 were tested. Diluted phage suspensions ($10^2$ – $10^9$ PFU/mL) were mixed with exponentially growing host cultures ($10^7$ CFU/mL) at a 1:1 volume ratio (50 μL phage + 50 μL host) in 990 μL SM Buffer and incubated at 37 °C and 150 rpm for 3.5 h. The lysates were centrifuged (12,000 × g, 4 °C, 3 min), and the supernatants were filtered through 0.22 μm membranes. Phage titers (PFU $mL^{-1}$) were determined using the double agar overlay plaque assay, and the MOI that produced the highest PFU value was designated as the optimal multiplicity of infection.

**Supplementary Text 2. Details of crystal violet staining and biofilm quantification.**

At designated time points, well contents were discarded, followed by 2 washes with PBS and complete air-drying. Biofilms were then stained with 1.0 mL of 0.1% (w/v) crystal violet solution for 10 min at ambient temperature. Excess stain was removed, and wells were rinsed with 1.0 mL PBS until effluent ran clear, then air-dried once more. Bound dye was subsequently extracted by adding 1.0 mL of 30% (v/v) glacial acetic acid with orbital shaking for 15 min. The resulting solutions were diluted 1:10, and optical density was determined at 595 nm using a microplate spectrophotometer.
